# First record of *Tyrodes* Raffray (Coleoptera, Staphylinidae, Pselaphinae) in China, with description of *T. jenisi* sp. n. from Yunnan Province

**DOI:** 10.3897/zookeys.301.4911

**Published:** 2013-05-17

**Authors:** Zi-Wei Yin, Li-Zhen Li

**Affiliations:** 1 Department of Biology, College of Life and Environmental Sciences, Shanghai Normal University, 100 Guilin Road, Shanghai, 200234, P. R. China

**Keywords:** *Tyrodes jenisi* Yin & Li, sp. n., of the pselaphine tribe Tyrini, from Yunnan, Southwest China is described, illustrated and distinguished from allied species mainly using aedeagal characters. This represents the first record of *Tyrodes* in China. Staphylinidae, Pselaphinae, taxonomy, *Tyrodes*, new species, China

## Introduction

The small genus *Tyrodes* Raffray currently contains six valid species scattered in the Oriental (5 spp.) and Northeast Palaearctic (1 sp.) regions: *Tyrodes histrio* (Schaufuss, 1887) (Sri Lanka, type species), *Tyrodes championi* (Jeannel, 1960) (India), *Tyrodes clavatus* (Raffray, 1895) (Singapore), *Tyrodes janetscheki* Besuchet, 1970 (Nepal), *Tyrodes setosus* Jeannel, 1957 (Vietnam), and *Tyrodes segrex* Kurbatov, 1990 (Russian Far East) (Raffray 1895, 1908; Newton and Chandler 1989; Hlaváč and Chandler 2005). *Tyrodes* is allied to the Holarctic and Oriental *Tyrus* Aubé by sharing a similar general habitus, and similar forms of the maxillary palpi and pronotum. The two genera can be separated by the presence of an indistinct frontal fovea, the abdominal tergite IV (first visible tergite) being longer than tergite V, and the aedeagus being stouter and has a short median lobe in *Tyrodes*, while *Tyrus* has a distinct frontal fovea, the tergite IV is subequal to tergite V, and the aedeagus is more slender and bears a longer median lobe.

In this paper, we report a new species of *Tyrodes* from Yunnan, Southwest China. A diagnosis, a description, and illustrations of male diagnostic features are provided. This also represents the first record of the genus in China.

## Material and methods

The holotype is housed in the private collection of Peter Hlaváč (pcPH), and will eventually be deposited in the National Museum of Natural History, Prague (NMPC)

The collection data of the referred material are quoted verbatim. A slash (/) is used to separate lines on the same label, and a double slash (//) is used to separate different labels.

All measurements are in millimeters. The following acronyms are applied: **AL**–length of the abdomen along the midline; **AW**–maximum width of the abdomen; **BL**–length of the body (= HL + PL + EL + AL); **EL**–length of the elytra along the sutural line; **EW**–maximum width of the elytra; **HL**–length of the head from the anterior clypeal margin to the occipital constriction; **HW**–width of the head across eyes; **PL**–length of the pronotum along the midline; **PW**–maximum width of the pronotum.

## Description of new species

### Tyrodes jenisi

Yin & Li sp. n.

urn:lsid:zoobank.org:act:01240E37-DF20-4B5F-99F9-BF8C7DA6A42E

http://species-id.net/wiki/Tyrodes_jenisi

Fig. 1

#### Type material

(1 ♂). Holotype: ♂, labeled ‘CHINA: Yunnan / Pass SW from Baoshan / Gaoligong Shan / 4–8.VI.2006, Jeniš lgt. // HOLOTYPE [red] / *Tyrodes jenisi* / sp. n., Yin & Li / det. 2013.’ (pvPH).

#### Description.

**Figure 1.**
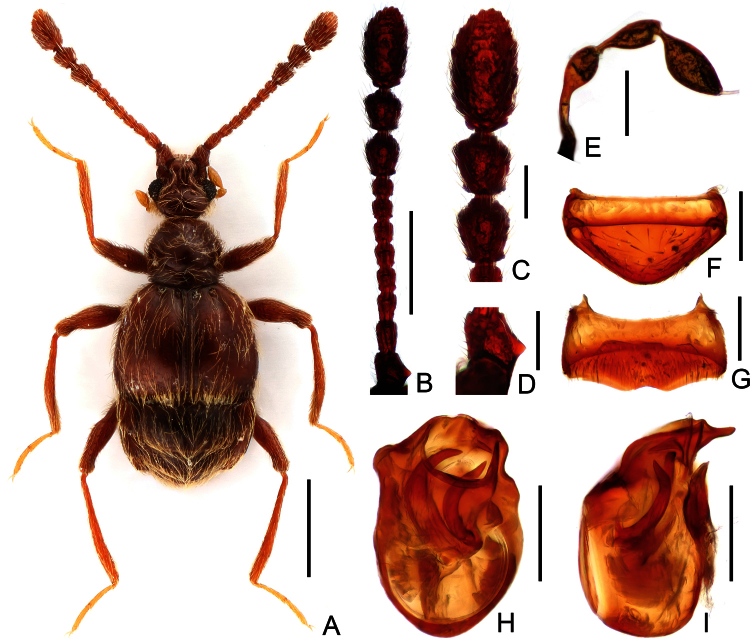
*Tyrodes jenisi*, male. **A** dorsal habitus **B** antenna **C** antennal club, enlarged **D** scape, enlarged **E** maxillary palpus **F** tergite VIII **G** sternite VIII **H** aedeagus, in dorsal view **I** same, in lateral view. Scales (mm): **A** = 0.5; **B** = 0.2; **C–I** = 0.1.

Male (Fig. 1A). Length 1.84 mm. Head about as long as wide, HL 0.37 mm, HW 0.36 mm; eyes each composed of about 35 facets; maxillary palpi as in Fig. 1E; antennae (Fig. 1B) elongate, scapes (Fig. 1D) triangularly projecting basolaterally, antennomeres II–VIII successively shorter; terminal three antennomeres enlarged (Fig. 1C). Pronotum about as long as wide, PL 0.39 mm, PW 0.38 mm, with rounded lateral margins, evenly narrowed apically at middle. Elytra wider than long, EL 0.55 mm, EW 0.77 mm. Legs lacking spines or projections. Abdomen broad at base and narrowed apically, AL 0.53 mm, AW 0.73 mm. Tergite VIII (Fig. 1F) and sternite VIII (Fig. 1G) transverse. Aedeagal length 0.24 mm, stout; with short, asymmetric median lobe (Figs 1H, I); endophallus composed of two sclerites curved to left.

Female. Unknown.

#### Comparative notes.

*Tyrodes jenisi* can be separated from all congeners, except for *Tyrodes clavatus*, by the distinct form of the aedeagus, as well as the consideration of distributional patterns.

*Tyrodes championi* is subequal in size (1.8 mm), but its aedeagal median lobe forms a distinct process at apex; *Tyrodes histrio* is smaller (1.5 mm), with the aedeagus being split at apex, and the endophallus with a long sclerite on the left side; *Tyrodes janetscheki* is greater in size (1.9 mm), its aedeagus has much more complicated structure of endophallus; *Tyrodes segrex* has a greater size as well (1.9–2.0 mm), and has clear different structure of aedeagal endophallus; *Tyrodes setosus* is subequal in size (1.8 mm), but the apical portion of aedeagal median lobe is curving to right, and has a strong apophysis on the right side. Aedeagus of *Tyrodes clavatus* has not been illustrated in any reference, but it is much smaller (1.4 mm), and is found in Singapore.

#### Comments.

In his world catalog of the genera of Pselaphidae, Raffray (1908) moved *Pselaphodes clavatus* Raffray, 1895 to *Tyrodes*, followed in the later Coleopterorum Catalogus (Raffray 1911). Jeannel (1957: 32) compared the new species *Tyrodes setosus* Jeannel with *Tyrodes clavatus* when treating the pselaphines collected from Tonkin, Vietnam by Albert de Cooman. Besuchet (1970: 316), Newton & Chandler (1989: 60) and Kurbatov (1990: 145) also suggested or mentioned the placement of *clavatus* in *Tyrodes*. In the recent catalog of Tyrini (Hlaváč and Chandler 2005), this placement was probably overlooked, the species was remained in the genus *Pselaphodes*. According to the original description, it is clear that *clavatus* belongs to *Tyrodes*.

#### Distribution.

Southwest China: Yunnan.

#### Etymology.

The new species is named after Ivo Jeniš (Náklo, Czech Republic), collector of the holotype.

## Acknowledgments

We thank Peter Hlaváč (Prague, Czech Republic) for providing the material used in this study. Alfred F. Newton kindly provided discussion on *Tyrodes clavatus*. Two anonymous reviewers are thanked for the critical comments on a previous draft. This study is supported by the National Science Foundation of China (No. 31172134), and Shanghai Normal University (Sk201242, DZL125).

## Supplementary Material

XML Treatment for Tyrodes jenisi
